# 

**Published:** 1860-08

**Authors:** 


**Index to Volume Fifth.**

The Index to the present Volume will be sent out with the first number of the Sixth Volume, commencing with September.

### TO CORRESPONDENTS.

By a recent change in the management of the Journal, its whole business affairs will hereafter come under the supervision of the Senior Editor, and it is therefore requested that ALL Communications, whether relating to the Editorial, Subscription, Advertising or Financial Departments, should be addressed to Jos. P. LOGAN, or Editors of Medical and Surgical Journal, Atlanta, Ga.

